# Involvement of Vanin-1 in Ameliorating Effect of Oxidative Renal Tubular Injury in Dahl-Salt Sensitive Rats

**DOI:** 10.3390/ijms20184481

**Published:** 2019-09-11

**Authors:** Keiko Hosohata, Denan Jin, Shinji Takai, Kazunori Iwanaga

**Affiliations:** 1 Education and Research Center for Clinical Pharmacy, Osaka University of Pharmaceutical Sciences, Osaka 569-1094, Japan; iwanaga@gly.oups.ac.jp; 2 Department of Innovative Medicine, Graduate School of Medicine, Osaka Medical College, Osaka 569-8686, Japan; pha012@osaka-med.ac.jp (D.J.); pha010@osaka-med.ac.jp (S.T.)

**Keywords:** sensitive biomarker, high-salt intake, hypertension, renal tubular damage

## Abstract

In salt-sensitive hypertension, reactive oxygen species (ROS) play a major role in the progression of renal disease partly through the activation of the mineralocorticoid receptor (MR). We have previously demonstrated that urinary vanin-1 is an early biomarker of oxidative renal tubular injury. However, it remains unknown whether urinary vanin-1 might reflect the treatment effect. The objective of this study was to clarify the treatment effect for renal tubular damage in Dahl salt-sensitive (DS) rats. DS rats (six weeks old) were given one of the following for four weeks: high-salt diet (8% NaCl), high-salt diet plus a superoxide dismutase mimetic, tempol (3 mmol/L in drinking water), high-salt diet plus eplerenone (100 mg/kg/day), and normal-salt diet (0.3% NaCl). After four-week treatment, blood pressure was measured and kidney tissues were evaluated. ROS were assessed by measurements of malondialdehyde and by immunostaining for 4-hydroxy-2-nonenal. A high-salt intake for four weeks caused ROS and histological renal tubular damages in DS rats, both of which were suppressed by tempol and eplerenone. Proteinuria and urinary N-acetyl-β-D-glucosaminidase exhibited a significant decrease in DS rats receiving a high-salt diet plus eplerenone, but not tempol. In contrast, urinary vanin-1 significantly decreased in DS rats receiving a high-salt diet plus eplerenone as well as tempol. Consistent with these findings, immunohistochemical analysis revealed that vanin-1 was localized in the renal proximal tubules but not the glomeruli in DS rats receiving a high-salt diet, with the strength attenuated by tempol or eplerenone treatment. In conclusion, these results suggest that urinary vanin-1 is a potentially sensitive biomarker for ameliorating renal tubular damage in salt-sensitive hypertension.

## 1. Introduction

A high-salt intake is one of the contributors to the development of hypertension. While most people remain normotensive if they have a high-salt diet due to the excretion of sodium in the urine, some people cannot freely excrete salt, thereby leading to an elevation in their blood pressure (BP) [1]. This phenomenon is defined as “salt sensitivity” [2], and accounts for 30–50% of hypertensive patients [3,4]. Furthermore, these salt-sensitive individuals have been shown to exhibit more cardiovascular [5] and renal [6] complications and have a greater mortality as compared to the salt-resistant hypertensive patients [7].

Salt and aldosterone have also been shown to synergistically act on the cardiovascular system [8]. The aldosterone/mineralocorticoid receptor (MR) system both dominates BP control as well as mediates the development of cardiovascular and renal disease, especially when there is an excess salt intake. Salt paradoxically activates the intrarenal renin-angiotensin system despite suppressing systemic renin release [9]. This occurs partly by enhancing the intrarenal generation of reactive oxygen species (ROS) [10,11]. Swei et al. [12] demonstrated that superoxide (O_2_^−^) production along with plasma hydrogen peroxide levels were significantly elevated and caused renal damage in Dahl salt-sensitive (DS) rats, which are used as a genetic model of salt-sensitive hypertension. Using the measurements obtained by the lucigenin chemiluminescence method, Trolliet et al. [13] demonstrated that the hypertensive nephropathy in DS rats was associated with increases in the renal tissue O_2_^−^ levels. In addition, in the presence of salt, aldosterone causes hypertension and renal damage via the MR, which influences a variety of different cell signaling such as oxidative stress, inflammation and renal fibrosis. These observations suggest that salt-dependent hypertension and nephropathy are accompanied by an increase in ROS. Thus, ROS play a central role in the development and progression of inflammatory responses in the progression of kidney disease. Based on these oxidative injuries, various agents have been shown to exhibit preventative effects in animal models. For example, eplerenone, an MR antagonist, exerts a beneficial effect via its anti-oxidative, anti-inflammatory and anti-fibrotic effects [14]. However, as of yet there have been no reports on any predictive biomarkers for the treatment effect. If such a biomarker were to exist, this could be helpful for use as a biomarker-adjusted treatment in a personalized medical regimen. As a result, such a marker could be used to help to minimize the required treatment and predict the response to the therapy, thereby allowing for adjustments to the treatment regimen on an individual patient basis.

Previously, we demonstrated that there was elevation of urinary vanin-1, which is a tissue sensor for oxidative stress, prior to urinary increases of traditional biomarkers such as N-acetyl-β-D-glucosaminidase (NAG), serum creatinine or blood urea nitrogen in rats with nephrotoxicant- and drug-induced renal tubular injury [15,16] and in cancer patients treated with cisplatin [17]. Vanin-1 is a glycosylphosphatidylinositol-anchored cell-surface protein [18,19] and plays a role in the oxidative stress response. Vanin-1 hydrolyzes pantetheine to pantothenic acid (vitamin B5) and cysteamine. Subsequently, cysteamine, which is a low-molecular thiol, is then converted to cystamine and inhibits γ-glutamylcysteine synthetase (γGCS), the rate-limiting enzyme of glutathione synthesis [20]. *Vanin-1* knockout mice, which lack free cysteamine in their tissues, have been shown to be resistant to oxidative stress as well as down-regulated tissue inflammation, thereby leading to lower oxidative tissue damage that is associated with the subsequent survival of these animals when exposed to stress [21].

We then used DS rats to test the hypothesis that vanin-1 is involved in ameliorating effect of renal tubular oxidative injury by administering a superoxide dismutase mimetic, tempol and a MR antagonist, eplerenone. 

## 2. Results

### 2.1. Effect of Tempol and Eplerenone on Systolic BP

All animals completed the study protocol. Male Dahl salt-resistant (DR) rats received a diet containing normal salt (N, 0.3% NaCl; DR/N) or high salt (H, 8% NaCl; DR/H) for four weeks, while the DS rats received a diet containing normal salt (DS/N), high salt (DS/H), high salt plus tempol (DS/H + tempol), or high salt plus eplerenone (DS/H + eplerenone) for four weeks.

As shown in Table 1, four weeks of salt feeding significantly elevated the systolic BP (SBP) in DS rats (160.8 ± 9.2 mmHg vs. 124.5 ± 2.4 mmHg), which was then suppressed by treatment with tempol (121.2 ± 7.4 mmHg) or eplerenone (132.2 ± 3.4 mmHg). In contrast, there were no significant changes observed after the salt feeding in the DR rats for the SBP, body weight or left kidney weight.

### 2.2. Effect of Tempol and Eplerenone on Renal Damage

After four-week feeding of high-salt diet, the kidney weight to body ratios of the DS/H rats were significantly higher than those observed for the DS/N rats.

While tempol treatment resulted in almost the same values for the kidney weight to body ratios in the DS/H rats, eplerenone treatment significantly suppressed the increase of the kidney weight to body ratios in the DS/H rats.

**Figure 1:**
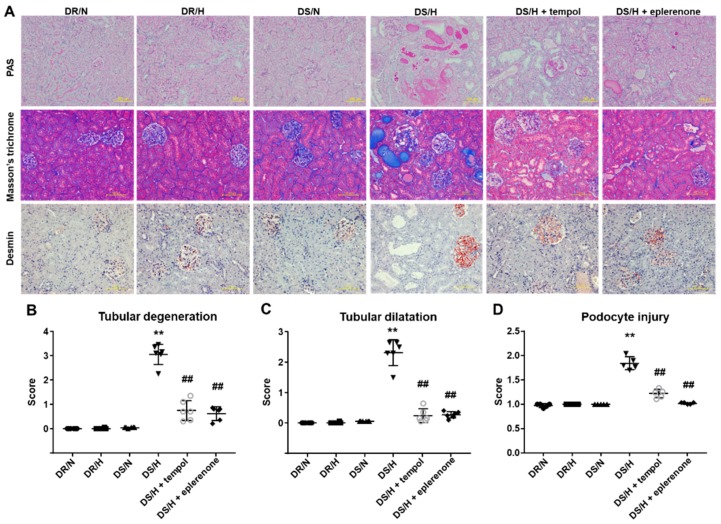
Histopathological examination of kidney tissues. Representative photomicrographs of periodic acid-Schiff (PAS), Masson’s trichrome staining and immunostaining for desmin of the kidney cortical regions (**A**). Tubular degeneration and dilatation were assessed using a semiquantitative score from 0 to 4, with 0 representing no damage and 4 representing severe damage (**B**,**C**). Podocyte injury marker desmin was assessed using a semiquantitative score from 0 (0%) to 4 (51% to 75%) for each animal (average per animal from 30 selected glomeruli) (**D**). Values are represented as means ± SE (*n* = 5–6). **, *p* < 0.01 vs. DS/N rats. ^##^, *p* < 0.05 vs. DS/H rats. Scale bar, 100 μm.

The renal histological evaluations with PAS staining in DR/N, DR/H and DS/N rats showed intact or very slight renal tubular damage. In contrast, DS/H rats exhibited severely damaged renal tubules, which were characterized by degeneration and dilatation, with many vacuolated tubules also observed (Figure 1A). Scoring of the degeneration and dilation confirmed these findings (Figure 1B,C). To evaluate the presence of podocyte injury, we performed immunohistochemistry of desmin, a conventional podocyte injury marker. Signals were few detected in the glomeruli of DR/N, DR/H and DS/N rats; whereas multiple glomeruli were positive for desmin in DS/H rats. These signals were attenuated by treatment of tempol and eplerenone (Figure 1D). In addition, Masson’s trichrome staining revealed that there were collagen deposits (stained blue) around the renal tubules in DS/H rats, whereas the DR/N, DR/H and DS/N rats exhibited a normal distribution of collagen fibers. Concurrent administration of tempol or eplerenone ameliorated these tubular changes and fibrosis.

### 2.3. Evaluation of Renal Tubular Injury by Traditional and Newly Developed Biomarkers

**Figure 2:**
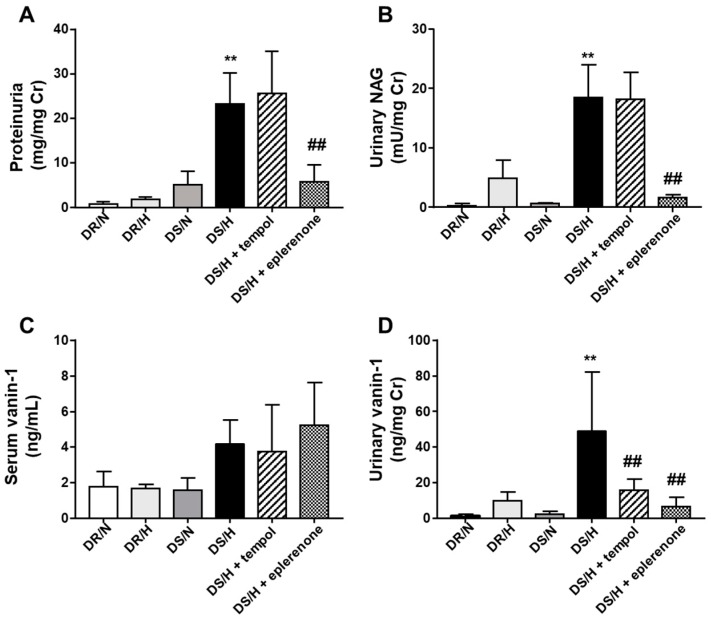
Evaluation of traditional or newly developed biomarkers. At four weeks after normal-salt or high-salt diets, 12 h urine or serum samples were collected for each group (**A**–**D**). Concentrations of proteinuria, urinary NAG, and urinary vanin-1 were normalized to the urinary creatinine concentration. Values are presented as means ± SE (*n* = 6). **, *p* < 0.01 vs. DS/N rats. ^##^, *p* < 0.01 vs. DS/H rats.

Urinary excretion of total protein was significantly higher in the DS/H rats (23.3 ± 2.8 mg/mg Cr) as compared to the DS/N (4.1 ± 0.9 mg/mg Cr), DR/H (1.8 ± 0.2 mg/mg Cr) and DR/N (0.7 ± 0.2 mg/mg Cr) rats. Eplerenone significantly reduced the urinary excretion of total protein in DS/H (5.8 ± 1.5 mg/mg Cr), but not tempol (25.6 ± 3.9 mg/mg Cr) rats (Figure 2A). Similarly, after four weeks of a high-salt diet, the DS/H rats (18.5 ± 2.2 mU/mg Cr) exhibited a higher urinary NAG as compared to the DS/N (0.7 ± 0.04 mU/mg Cr), DR/N (0.2 ± 0.2 mU/mg Cr) and DR/H (4.9 ± 1.2 mU/mg Cr) rats. Eplerenone (1.6 ± 0.2 mU/mg Cr) markedly and significantly reduced the urinary NAG, whereas tempol (18.1 ± 1.9 mU/mg Cr) did not significantly decrease the urinary NAG (Figure 2B). There were no significant differences observed between the groups for the serum vanin-1 (Figure 2C). However, urinary vanin-1 was significantly higher in the DS/H (49.0 ± 13.6 ng/mg Cr) than that in the DS/N (2.3 ± 0.7 ng/mg Cr) rats, with both eplerenone (6.7 ± 2.1 ng/mg Cr) and tempol (15.7 ± 2.5 ng/mg Cr) treatments decreasing the levels (Figure 2D).

**Figure 3:**
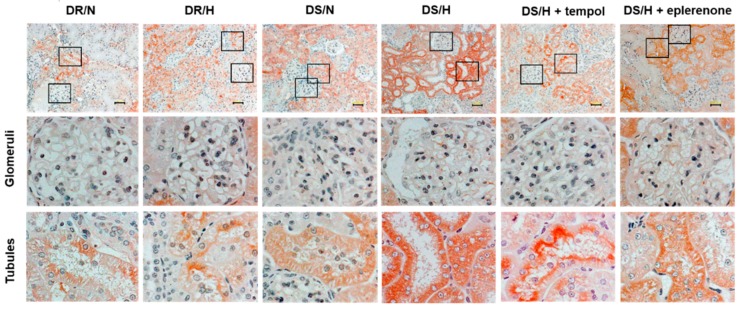
Immunohistochemistry of vanin-1 in rat kidney tissues. Representative images of kidney cortical regions. Magnified views of glomeruli and tubules are from the indicated black boxes. Vanin-1 expression was localized both around the injured tubules and within a subset of injured tubules, but not in the glomeruli. Scale bar, 50 μm.

Immunohistochemical analysis revealed that vanin-1 was localized in the proximal tubules, but not the glomeruli (Figure 3). The intensity of vanin-1 was obviously strong in the DS/H rats and was observed to be attenuated in the tempol- or eplerenone-treated rats.

### 2.4. Effect of Eplerenone and Tempol on Oxidative Stress

**Figure 4:**
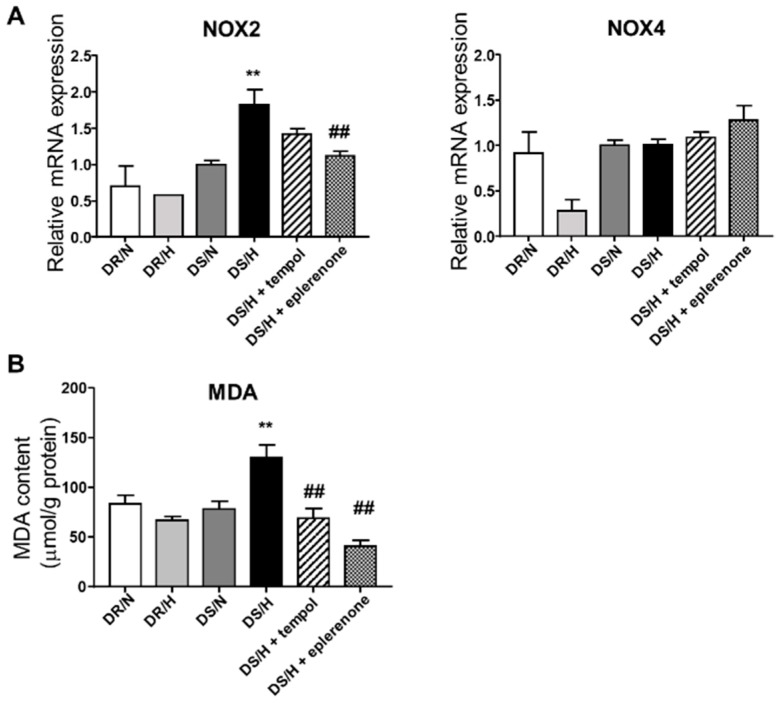
Evaluation of oxidative stress. Analysis of the renal gene expression of *Nox2* and *Nox4* (**A**) and MDA content (**B**). MDA, malondialdehyde. Values are presented as means ± SE (*n* = 6). **, *p* < 0.01 vs. DS/N rats. ^##^, *p* < 0.01 vs. DS/H rats.

Oxidative stress is thought to be an important contributor to the pathogenesis of renal disease. To assess the involvement of oxidative stress in the progression of the renal injury, we quantified mRNA expressions of nicotinamide adenine dinucleotide phosphate (NAD(P)H) oxidase, (Nox). In DR rats, high-salt loading did not increase the expression of *Nox2* mRNA in the renal cortical tissues. In contrast, DS/H rats exhibited a significantly increased expression of *Nox2* mRNA as compared to the DS/N rats (Figure 4A). Eplerenone significantly decreased *Nox2* mRNA expression. Furthermore, the MDA results revealed that a high-salt diet caused significantly elevated oxidative stress, although this elevation was inhibited by tempol and eplerenone (Figure 4B).

**Figure 5:**
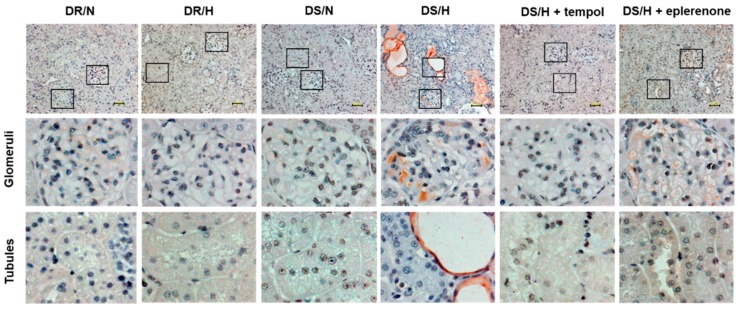
Immunohistochemical analysis of oxidative stress. Representative immunostaining of 4-hydroxy-2-nonenal (4-HNE) in the kidney of rats. Magnified views of glomeruli and tubules are from the indicated black boxes. 4-HNE was positive in the injured tubules of the rats. Scale bar, 50 μm.

Immunohistochemical analysis showed that there was an increased expression of 4-HNE, which is an oxidative stress marker, in damaged tubules with prominent expression in the renal tubular cells (Figure 5). Of note, the tubular staining level for 4-HNE appeared to be stronger in the DS/H than that in the DS/N, and it was attenuated in eplerenone- or tempol-treated rats.

## 3. Discussion

In the current study, urinary vanin-1 but not the traditional renal marker, proteinuria or urinary NAG, reflected the ameliorating effect associated with the administration of tempol or eplerenone in DS/H rats. Vanin-1 was expressed in the renal tubules and not in the glomeruli when there was oxidative injury in the salt-sensitive model of hypertension, with the marker of oxidative stress also co-localized in the renal tubules. In addition, treatment with eplerenone markedly reduced the mRNA expression of Nox2, which is NAD(P)H oxidase, in the renal tubules while 4-HNE staining weakened and was accompanied by ameliorated tubular injury in these rats. To the best of our knowledge, this is the first study to show that urinary vanin-1 is an early biomarker for the ameliorating effects seen during the progression of renal injury due to oxidative stress.

It is well known that salt loading suppresses renin release from the renal juxtaglomerular apparatus, thereby causing a reduction in the systemically generated angiotensin II. However, a basic study has demonstrated that in spite of the suppression of circulating angiotensin II under salt loading, there was elevation of the salt-stimulated locally intrarenal renin-angiotensin-aldosterone system (RAAS) and angiotensin II content of the proximal tubular fluid [21]. Therefore, it is possible that the stimulated intrarenal RAAS might be involved in renal tubular damage under salt-loading conditions. Aldosterone has been demonstrated to play a major role in regulating extracellular fluid volume, maintaining sodium and potassium balance [22] and independently mediating kidney injury and the progression of chronic kidney disease [23]. In addition, aldosterone is also involved in the genomic or non-genomic pathway [24], with the genomic effects generally considered to be mediated by the MR, whereas the nongenomic effects are not blocked by inhibitors of transcription [25]. It has been further shown that aldosterone and the MR are implicated in the activation of inflammation, remodeling and fibrosis in several target organs [26]. As aldosterone increased the macrophage superoxide release and macrophage-mediated low-density lipoprotein oxidation [27], this suggests that aldosterone directly induces oxidative stress. In the present study, we found that the suppressive effect of eplerenone on oxidative injury was greater than that observed for tempol. This might be possible due to the involvement of eplerenone in the suppression of both the genomic and nongenomic effects.

It is well-known that the main source of O_2_^−^ is Nox. Nox is present in the renal cortex, the medulla, as well as in blood vessels [28,29]. Among the different members of the Nox family, Nox2 and Nox4 are abundantly expressed in the kidney of rats [30,31,32,33,34]. The Nox2 isoform of NAD(P)H generates primarily O_2_^−^ [30,35]. On the other hand, the Nox4 isoform of NAD(P)H oxidase primarily generates H_2_O_2_ [35,36]. It has been previously demonstrated that after salt loading, oxidative stress causes hypertension and renal damage [37]. With hypertension, ROS production directly causes endothelial dysfunction and changes the contractility, thereby leading to vascular remodeling. In the central nervous system, overproduction of ROS leads to increased sympathetic outflows and elevated BP [38,39]. When there is renal damage, suppression of NAD(P)H oxidase activity by renal denervation reduces glomerular injury in DS rats [40]. ROS can trigger an inflammatory response through activation of the tumor necrosis factor-α (TNF-α) pathway [41]. TNF-α promotes monocyte chemotaxis towards mesangial cells through the regulation of the expression of monocyte chemoattractant protein-1 (MCP-1) via a Rho-kinase/p38 mitogen-activated protein kinase (MAPK)-dependent pathway [42]. In addition, ROS activate p38MAPK and the transcription factor, nuclear factor kappa B (NF-κB), which leads to proinflammatory cytokine release and inflammatory cell accumulation in the kidney [43,44]. Thus, it is possible that sustained inflammatory responses could contribute to the progression of renal injury [45].

Most of the previous reports on the association of oxidative stress with renal injury involved glomerular injury. However, tubular injury also affects the glomerular filtration function via the tubuloglomerular feedback [46]. In addition, renal tubules play an important role in the maintenance of body fluid homeostasis and the defense of the body against toxic reactions via absorption and secretion of various xenobiotics and endogenous compounds. Thus, it is important to be able to detect renal injury at an early stage, start intervention, and follow the ameliorating effect via the use of renal tubular biomarkers.

In the present study, we found that the oxidative injury was localized in the renal proximal tubules, but not in the glomeruli. This is consistent with the previous report that vanin-1 was induced by ROS in vivo [34]. Of particular note is the finding that the immunohistochemical signals were diminished after the administration of tempol and eplerenone, with urinary vanin-1 reflecting the amelioration observed after both the tempol and eplerenone treatments. This is in contrast to the traditional biomarkers, proteinuria and urinary NAG, which reflect the renal histology after the administration of eplerenone only. While proteinuria is used as a marker of glomerular injury rather than renal tubular injury, NAG has been often used as a marker of renal tubular injury. In the present study, there was a significant decrease in the urinary NAG after a treatment of eplerenone (*p* < 0.01) but not tempol. However, our histological analysis revealed that renal tubular injury was obviously suppressed by tempol, with the renal score of renal degeneration and dilatation significantly decreased by tempol. In contrast, since urinary vanin-1 appeared to reflect the effect associated with tempol, this suggests that urinary vanin-1 could be a sensitive renal tubular biomarker of the renal effect of the treatment.

## 4. Materials and Methods

### 4.1. Experimental Protocol

All animal procedures were approved on 11 October 2018 by the Committee of Animal Use and Care of Osaka Medical College (No. 30108) and performed in accordance with the Guidelines for Animal Research. Male DR and DS rats (Japan SLC, Shizuoka, Japan) were obtained at 6 weeks of age and maintained under specific pathogen-free conditions with controlled temperature and humidity with a 12-h light–dark cycle. All rats were fed a regular diet (CE-2; CLEA Japan, Tokyo, Japan) and given water ad libitum before their use in the experiment. After 1 week, DR rats were divided into 2 groups in which one group received a diet containing normal salt (N; 0.3% NaCl, CLEA Japan) (DR/N; *n* = 6) and the other a diet with high salt (H; 8% NaCl; CLEA Japan) (DR/H; *n* = 6). The 24 DS rats were divided into 4 groups. One group was fed a normal-salt diet (DS/N; *n* = 6), while the rats in other 3 groups were fed a high-salt diet under the administration of tempol (Sigma Co. St Louis, MO, USA; 3 mmol/L in drinking water; *n* = 6), eplerenone (gifted by Pfizer, New York, NY, USA; 100 mg/kg/day; *n* = 6) or vehicle (DS/H; *n* = 6). Once a week, rats were placed in metabolic cages for a 12-h period in order to collect urine samples. All urine samples were stored at −80 °C until use. After 4 weeks, SBP was monitored at frequent intervals under a prewarmed conscious state, using a tail-cuff microsensor device (model MK-2000ST; Muromachi Kikai, Tokyo, Japan), during which the rats were anesthetized with isoflurane to obtain blood and kidney tissues.

### 4.2. Histological Analysis

After kidney tissues were fixed with Carnoy Solution (Muto Pure Chemicals Co., Ltd., Tokyo, Japan) for 24 h and embedded in paraffin, the sections (4 μm) were stained with periodic acid-Schiff (PAS) and Masson’s trichrome as previously detailed [47]. Tubular degeneration and tubular dilatation were graded as 0–4 (grade 0, normal; grade 1, mild; grade 2, moderate; grade 3, severe; and grade 4, very severe) using a semiquantitative scoring. An average score of 20 view-fields at ×200 magnification per animal was calculated for each group.

### 4.3. Immunohistochemical Analysis

The sections were deparaffinized with lemosol (Wako Pure Chemicals, Osaka, Japan), placed in a series of graded ethanol and washed with PBS. The sections were then soaked in 3% H_2_O_2_ in methanol at room temperature for 5 min to inhibit endogenous peroxidase activity. All sections were incubated with protein-blocking solution (Dako, Carpinteria, CA, USA) for 5 min to suppress non-specific binding. Following another PBS wash, the sections were then incubated with anti-desmin antibody (Dako) (1:100 dilution), anti-vanin-1 antibody (Uscn Life Science Inc., Wuhan, China) (1:50 dilution) or anti-4-hydroxy-2-nonenal (4-HNE) antibody (JaICA, Shizuoka, Japan) (1:100 dilution) at 4 °C overnight. Subsequently, the slides were then incubated with biotin-conjugated secondary antibody (LSAB 2 Kit/HRP; Dako) for 30 min after washing with PBS. Next, the sections were incubated with avidin-biotin-peroxidase complex (Dako) for 30 min, washed with PBS and incubated with 3-amino-9-ethylcarbazole (AEC) (AEC^+^ High Sensitivity Substrate Chromogen, Ready-to-Use, Dako). The slides were washed in running water, counterstained with hematoxylin and mounted with cover glasses.

To quantify podocyte injury, 30 glomeruli from each animal were examined for the presence of the podocyte injury marker desmin. The percentage area of desmin per glomerulus was calculated with the WinROOF2015 (MITANI Corporation, Fukui, Japan) and glomerular desmin staining was scored as follows: Signal area in the glomerulus was 0, 0%; 1+, 1% to 25%; 2+, 26% to 50%; 3+, 51% to 75%; and 4+, 76% to 100% [48]. The average of the score was calculated for each animal.

### 4.4. Laboratory Measurements

After urine and blood samples were centrifuged at 1000× *g* for 10 min, supernatant and serum samples were obtained. Urinary creatinine concentration was measured by the Jaffe method using a commercial kit (Wako Pure Chemical Industries, Osaka, Japan). Vanin-1 was measured using an enzyme-linked immunosorbent assay (ELISA) kit (Uscn Life Science Inc., Wuhan, China). Urinary protein and NAG were measured by a capillary electrophoresis-based method and a colorimetric method, respectively (SRL, Tokyo, Japan).

### 4.5. Measurement of Malondialdehyde (MDA)

Using the kidney tissue homogenate, the analysis of MDA, a product of lipid peroxidation, was conducted using a commercial kit (Cayman Chemical Company, Ann Arbor, MI, USA), which is based on the principle of quantifying the product from the reaction of MDA and thiobarbituric acid according to the manufacturer’s instructions.

### 4.6. RNA Extraction and Real-Time Quantitative PCR

Total RNA was isolated from the kidney tissues using an RNeasy Mini Kit (Qiagen, Valencia, CA, USA) and then reverse-transcribed using a PrimeScript RT Reagent Kit (Takara Bio, Otsu, Japan). Gene expression was analyzed by a real-time quantitative PCR, performed using the Applied Biosystems StepOnePlus Real-Time PCR system (Life Technologies, Carlsbad, CA, USA). Specific sets of primers and TaqMan probes were obtained from Life Technologies. To control for variation in the amount of cDNA available for PCR in the different samples, expression of target sequences was normalized to that of an endogenous control, Glyceraldehyde-3-phosphate dehydrogenase (*GAPDH*). The GenBank accession number, assay ID and target exon were NM_023965.1, Rn00576710_m1 and 3–4 for Cybb; NM_053524.1, Rn00585380_m1 and 12–13 for Nox4; NM_017008.3, Rn99999916_s1 and 1–1 for rat *GAPDH*, respectively. Data were analyzed using the comparative threshold cycle method.

### 4.7. Statistical Analysis

Data are expressed as the means ± SE. Data were compared using one-way ANOVA followed by Tukey’s post hoc analysis. A value of *p* < 0.05 was considered statistically significant. All statistical analyses were conducted with GraphPad Prism, version 4.03 (GraphPad Software, Inc., San Diego, CA, USA).

## 5. Conclusions

In order to initiate appropriate therapeutic intervention, detection of renal tubular damage at an early stage is clinically important. In addition, it is also essential to be able to estimate preventative or treatment effects using a sensitive biomarker. The present study provides a hypothesis that urinary vanin-1 might be a potentially sensitive biomarker of the ameliorating effect of drugs for oxidative tubular damage, especially in the salt-sensitive model of hypertension. To further address the hypothesis, additional prospective clinical studies will need to be undertaken.

## Figures and Tables

**Table 1:** Parameters at four weeks after the procedures in DR and DS rats.

Parameters	DR Rats		DS Rats			
	Normal Salt (DR/N)	High Salt (DR/H)	Normal Salt (DS/N)	High Salt (DS/H)	High Salt + Tempol (DS/H + Tempol)	High Salt + Eplerenone (DS/H + Eplerenone)
SBP, mmHg	101.7 ± 4.5	110 ± 2.6	124.5 ± 2.4	160.8 ± 9.2 ^aa^	121.2 ± 7.4 ^bb^	132.2 ± 3.4 ^b^
BW, g	364.8 ± 4.4	354.5 ± 3.6	336.5 ± 3.3	316 ± 5.4 ^aa^	308.2 ± 6.9	296 ± 7.4 ^bb^
Left KW, mg/g BW	3.6 ± 0.1	3.3 ± 0.06	3.9 ± 0.09	5.2 ± 0.13 ^aa^	5.4 ± 0.16	4.2 ± 0.06 ^bb^

BW, body weight; KW, kidney weight; SBP, systolic blood pressure. Data are expressed as means ± SE. DR, Dahl salt-resistant; DS, Dahl salt-sensitive. ^aa^ *p* < 0.01 vs. same strain on a normal-salt diet. ^b^ *p* < 0.05, ^bb^ *p* < 0.01 vs. vehicle.

## Abbreviations

BP	Blood pressure
DR rats	Dahl salt-resistant rats
DS rats	Dahl salt-sensitive rats
GAPDH	glyceraldehyde-3-phosphate dehydrogenase
GSH	glutathione
γGCS	γ-glutamylcysteine synthetase
4-HNE	4-hydroxy-2-nonenal
MAPK	mitogen-activated protein kinase
MR	mineralocorticoid receptor
MDA	malondialdehyde
NAG	N-acetyl-β-D-glucosaminidase
NOX	NADPH oxidase
O_2_^−^	superoxide
ROS	reactive oxygen species
RAAS	renin-angiotensin-aldosterone system
